# Maximum temperature drove snow cover expansion from the Arctic, 2000–2008

**DOI:** 10.1038/s41598-017-15397-3

**Published:** 2017-11-08

**Authors:** Yi Lin, Miao Jiang

**Affiliations:** 1 School of Earth and Space Sciences, Peking University, Beijing, 100871 China; 2 Institute of Mineral Resources Research, China Metallurgical Geology Bureau, Beijing, 100025 China

## Abstract

Investigating annual phenology of snow cover around the circumpolar Arctic is of significance for better grasping the effect of environment variation on global climate change, and previous studies found that temperature is the kernel climate feature interlinking with snow onset. However, how temperature closely drives snow cover expansion has not been fully exploited. Our analysis based on the Special Sensor Microwave Imager (SSM/I) and Special Sensor Microwave Imager Sounder (SSMIS) (i.e., SSM/I-SSMIS) data during 2000–2008 showed that the snow onset date (Do) was primarily driven by the maximum temperature approximately at the 22^nd^ day in advance (termed as the optimal period, OP) in the Northern Hemisphere. Specifically, the spatial patterns of the Do trends are similar with the previous finding, e.g., east Europe and west Asia exhibiting the most notable Do delay, and the OP days in latitude show the principal trend of first decreasing and then increasing. These characteristics can be attributed to the variation of the maximum temperature feature in latitude. Overall, the derivation of the statistical rules of temperature driving snow cover expansion from the Arctic facilitates predicting the occurrence of snow and understanding various terrestrial processes.

## Introduction

Snow cover annually expanding from the Arctic along with winter in the Northern Hemisphere (NH) coming and next shrinking back along with summer returning regulates a large variety of natural processes on the Earth^1^. The processes involve land energy balance, climate, hydrology, biology, ecology, etc. Specifically, snow cover phenology (SCP) that can quantify this cyclical dynamics can modulate land surface reflectivity such as radiative forcing and albedo^2,3^, and its variations may lead to climate change^4^, e.g., significant undulations in land surface temperature (LST)^5^; SCP can also adapt or even decide the supply of water resources in particular terrestrial regions, and its abnormity may trigger hydrological extreme events^6^; SCP is strongly associated with some biological and ecological phenomena, and its fluctuations may imbalance the related global biological and ecological systems^7,8^; the most well-known case about the significance of snow cover is protecting crops from cold air in winter, and abnormal SCP, no doubt, means crop yield decreasing in the harvesting season^9^; in a broader sense, SCP controls soil thaw and freeze dates, and its irregularity may influence the seasonality in terrestrial ecosystems^10^; furthermore, learning SCP facilitates projecting future climate scenario and potential global change^11^. Hence, investigating the SCP rules and its driving rules is essential for wide-spread nature understanding.

Previous studies found that SCP in the NH has kept varying^3,12^ and its variations are related to different environmental factors^3,6^. The first aspect highlights uncovering the intrinsic rules for the purpose of projecting SCP in future. For instance, it was found that snow cover in the NH has shrunk significantly. This is evidenced by the findings that the annually-averaged snow duration days decreased obviously in the NH snow-covered regions during 2001–2014^3^ and the June snow cover extent decreased about twice as rapidly as the widely recognized September loss of sea ice extent (SIE) during 1979–2011^12^. Previous studies have also suggested that SCP may remarkably change locally or regionally, with delayed snowfall onset dates^13^, earlier snowmelt onset dates^14^, earlier snow end dates^13,15^, and synthetically, shortened snow duration days^13–16^. The second aspect emphasizes the potential causes of SCP variations. Previous studies have summarized that SCP is extremely sensitive to the changes in temperature and precipitation^3,15^, and recent studies, further, revealed that significant changes in the increased winter precipitation can also be induced by atmospheric circulation changes^17^ and human activity^18,19^. Such inferences can help people do better in managing water resources in balance, maintaining sustainable ecosystems and predicting catastrophic climate events.

However, previous endeavors mostly focused on induction of the teleconnections between SCP variations and different environmental factors, few on analysis of the environment-driving patterns. In the case of temperature, the quantitative correlations between summer temperature extremes and winter snow onset dates have been deduced^6,20,21^, whereas the statistical rules of the adjacent temperature variations driving snow cover expansions from the Arctic has not been fully revealed. Specifically, for each local region in the NH, how its snow onset date every year is decided by its “driving-temperature” conditions, such as the value of snow-triggering temperature (STT), the optimal period (OP, i.e., the temporal range from the STT-related date to the snow onset date)^22^, and the spatial heterogeneity, is unclear. In other words, people still have got few explicit ideas about what kinds of temperature features and how many days in advance to be able to predict the occurrences of snowfalls for different regions across the NH. Hence, the objective of this study is to investigate the spatial and temporal characteristics of the NH snow onset dates (Do) around the Arctic and to figure out its temperature-driving rules. This kind of knowledge is critical for both fundamentally improving land surface models^12^ to investigate the inherent mechanism of environment factors driving snow cover expansion and macroscopically projecting future climate situations^12^ and the feedbacks of snow cover change to permafrost and vegetation^23^.

The analysis was conducted based on the Special Sensor Microwave Imager (SSM/I) and the Special Sensor Microwave Imager Sounder (SSMIS) (i.e., SSM/I-SSMIS) dataset (2000–2008)^24^, which derived the necessitated variables of land surface temperature (LST) and snow cover status synchronously at the daily scale from the same set of satellite remote sensing data. This advantage supersedes the other snow datasets^25–29^, which gave the variable of snow cover status only or the variables of LST and snow cover status synchronously but at coarser temporal scales. Next, when conducting the analysis, we also assumed some other restrictions as the same as those in the previous studies^3,30,31^. First, in accordance to the common trait of snow cover in the NH showing seasonal cycles^3,30^, the snow cover accumulation season considered in this study was defined as the period from the previous year (*t*-1) August to the current year (*t*) July. Second, we defined Do as the first five consecutive days on which snow can be noticed to cover the ground surface in the snow cover accumulation season^3,31^. More details about the settings of the present study can refer to Methods.

## Results

### Spatial and temporal pattern of snow cover expansion

**Figure 1:**
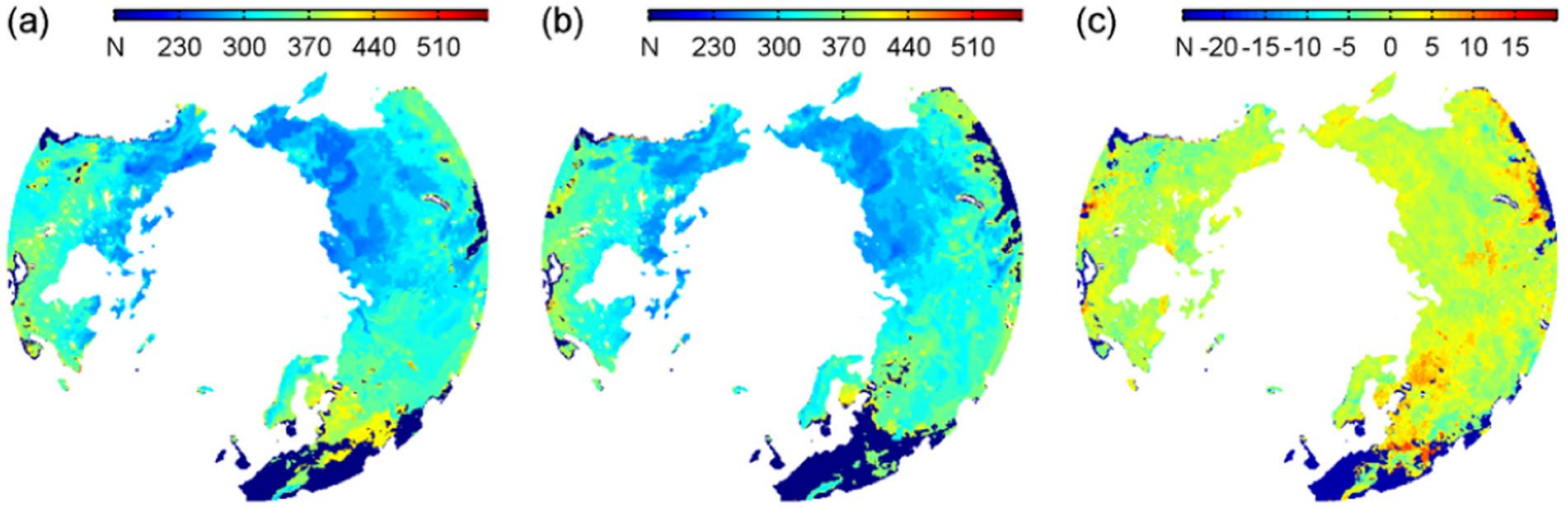
Illustration of snow onset date (Do) over the North Hemisphere (NH) (>45°N) between 2000 and 2001 (**a**) and between 2007 and 2008 (**b**) and snow Do slope (**c**) from 2000 to 2008 derived from the used dataset. (**a**–**c**) are mapped at a 25 × 25 km^2^ resolution. The figure was created using *Matlab* ^38^.

The resulting Do dates in the NH are annually illustrated in Fig. 1a and b, which present the spatial characteristics of snow cover expansion from the Arctic during 2000–2001 and 2007–2008, respectively. The results during 2001–2007 are shown in Supplementary Fig. S1. The Alaska and Russian Far East exhibited the earliest snow onset dates, the most obvious Do delay occurred over the East Europe and West Asia, and the West and Middle Europe underwent almost no real-sense snow covering, as defined in Introduction. From the circumpolar Arctic to the temperate zone, the snow onsets were gradually delayed in a general sense. The derived slopes of the Do values from 2000 to 2008 are shown in Fig. 1c, which does not demonstrate the same spatial pattern as the Do. Although there were some regions with extremely varying Do dates, the slopes over the principal parts of the NH were statistically ranging from 0 days/year to 3 days/year (within 90% confidence level), which were consistent with the findings about the snow Do with notable delays, e.g., 17.36 ± 6.67 days for the period of 2001–2014^3^.

**Figure 2:**
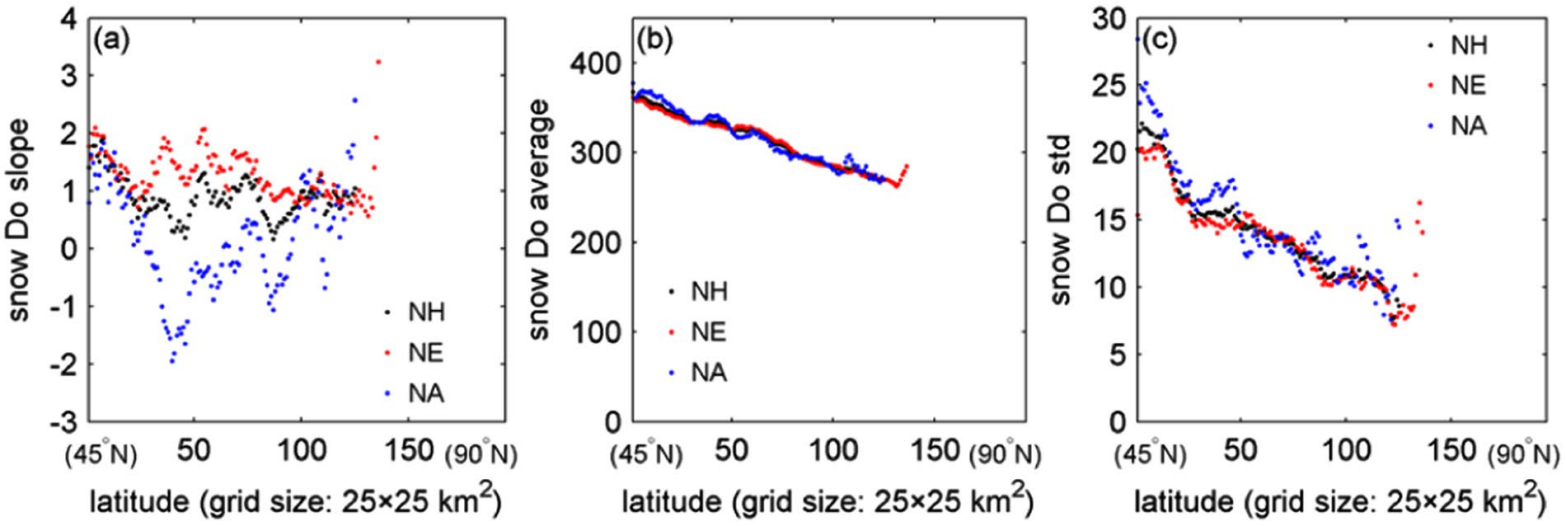
Change of the snow onset date (Do) slope (**a**), snow Do average (**b**) and snow Do standard deviation (std) (**c**) in latitude over the North Hemisphere (NH), North America (NA), and North Eurasia (NE) (>45°N) from 2000 to 2008 derived from the used dataset, respectively. The figure was created using *Matlab* ^38^.

The latitudinal changes of the snow Do slope, Do average and Do standard deviation (std) are listed in Fig. 2a,b, and c, respectively. The Do slope for the whole NH varied in latitude, and its value range fits the finding that the Do dates in most parts of the NH high latitudes (40–70°N) was delayed (e.g., 2.19 ± 1.63 days)^3^. The North Eurasia (NE) exhibited the similar pattern, but the North America (NA) behaved with difference. The central part of the NA in latitude showed a Do-advancing trend. This can be inferred from the result in the referenced study^3^, which, though, didn’t clarify this characteristic. The snow Do average and std both consistently decreased toward the Arctic. Specifically, larger the latitude, earlier the snow onset; closer to the Arctic, more stable the snow Do date in the same latitude. These deduced rules about the snow Do average and Do std work for the NH, NA, and NE all.

### Statistical rules in temperature driving snow cover expansion

**Figure 3:**
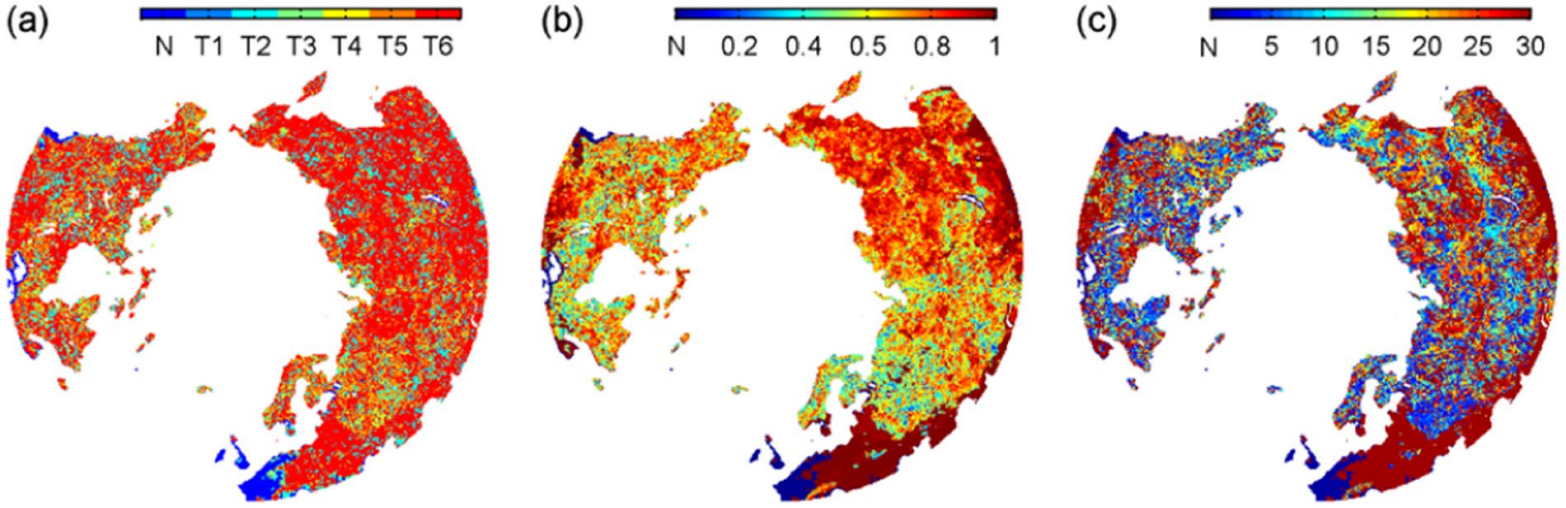
Distributions of the type (**a**), maximum correlation coefficient (r^2^) (**b**) and OP days (**c**) of the temperature parameters showing the highest correlations with the snow Do over the North Hemisphere (NH) (>45°N). (**a–c**) are mapped at a 25 × 25 km^2^ resolution. The figure was created using *Matlab* ^38^.

The correlations between the snow Do data and the prescribed six temperature parameters in the optimal cases are shown in Fig. 3. Figure 3a reveals the secret about which parameters best drive snow expansion in different NH grids. For most of the grids, the maximum temperature within the OP phase drives the expansion of NH snow cover. Figure 3b exposes the dependence of snow cover expansion on the optimal parameters. Asia and middle Europe show the stability, while north and east Europe and North America present relatively looser dependence. Figure 3c lists the numbers of the OP days. In a whole sense, larger the altitude, longer the OP. That is, the snow cover closer to the Arctic is more easily triggered by the temperature changes in the related past fewer days. But for the three characteristics (the type, maximum r^2^ and OP days) of interest, no consistent spatial patterns exhibit.

**Figure 4:**
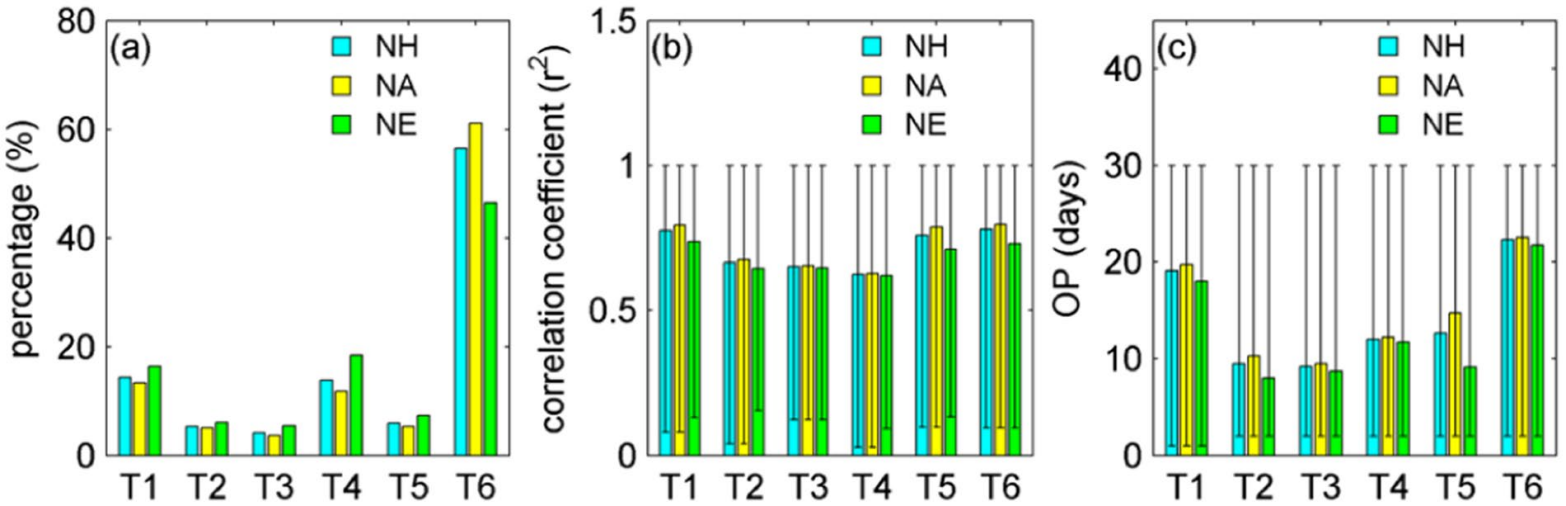
Statistics of the percentage (**a**), correlation coefficient (r^2^) (**b**) and OP days (**c**) of the optimal correlation cases for the six types of temperature parameters, separately in terms of North Hemisphere (NH), North America (NA), and North Eurasia (NE) (>45°N). The figure was created using *Matlab* ^38^.

The modes about temperature driving snow expansion over the NH (Fig. 3) are statistically analyzed, as listed in Fig. 4. For the NH and its two continents, the high temperature extreme in the OP phase played a leading role in driving snow expansion (totally ~58% of grids), far beyond the other five temperature parameters (Fig. 4a). Although the correlation coefficients cover a large value range from merely being larger than 0 to almost reaching 1 at the individual grid level, the average r^2^ values are satisfactory (Fig. 4b). The OP days also exhibit a large value range from one day to 30 days at the individual grid level, whereas their individual averages vary for different temperature parameters. The mean and maximum temperature relate to 18~23 OP days, while the other four parameters of the standard deviation, difference, slope and the minimum of the temperature values perform best with 7~15 OP days. Synthetically, the snow cover in the NH during 2000–2008 was primarily driven by the high temperature extreme in the adjacent 22^th^ day in advance, compared to the other five prescribed feature parameters.

**Figure 5:**
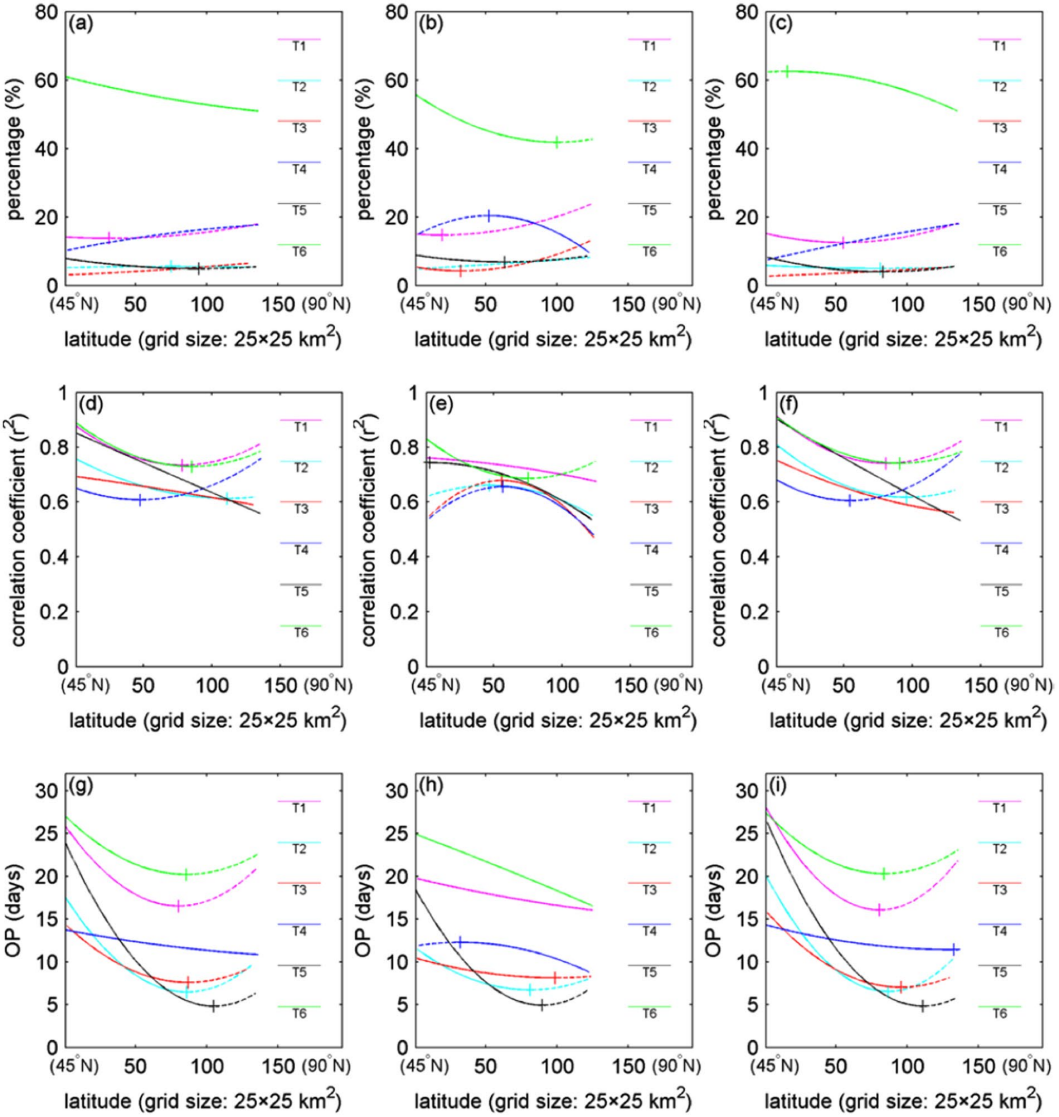
Change of percentage (**a**,**b** and **c**), correlation coefficient (r^2^) (**d**,**e** and **f**) and OP days (**g**,**h** and **i**) of the optimal correlation cases in latitude for the six types of temperature parameters. (**a**,**d** and **g**) North Hemisphere (NH), (**b**,**e** and **h**) North America (NA), and (**c,f** and **i**) North Eurasia (NE) (>45°N). The figure was created using *Matlab* ^38^.

The latitudinal changes of the percentages of the grids displaying the optimal correlations of the snow Do dates with the corresponding temperature parameters are shown in Fig. 5a–c for the NH, NA and NE, respectively. Their correlation coefficients r^2^ and OP days are listed in Fig. 5d–f and g–i (For more details about their derivations, the readers can refer to Supplementary Fig. S2). For the parameter of maximum temperature, its percentage over the whole NH takes the largest proportion and monotonously decreases towards the Arctic. But for the NA and NE, the trends both are varying but in contrast, i.e., decreasing to increasing and increasing to decreasing along with the latitude increasing, respectively. The other five temperature parameters proved to show turnings in latitude as well. The latitudinal changes of the correlation coefficients showed similar turnings, but their values all are high, mostly beyond 0.5. The latitudinal curves of the OP days also exhibited turnings for the NH and NE, i.e., the mainstream mode alters from decreasing to increasing along with the latitude increasing. However, the NA kept decreasing towards the Arctic. Overall, it can be noticed that the NA and NE demonstrate quite a lot of differences in the trends of these curves, and the whole performance in the NH is more like to those of the NE. The measures of temperature driving snow cover expansion from the Arctic were deduced as follow: along with the distance from the Arctic adding, the leading temperature parameter of maximum temperature played a strengthening, weakening to strengthening, and strengthening to weakening role for the NH, NA and NE, respectively; the related OP days kept to a decreasing to increasing, increasing, and decreasing to increasing trend, respectively.

## Discussion

### Spatial and temporal characteristics of the STT

**Figure 6:**
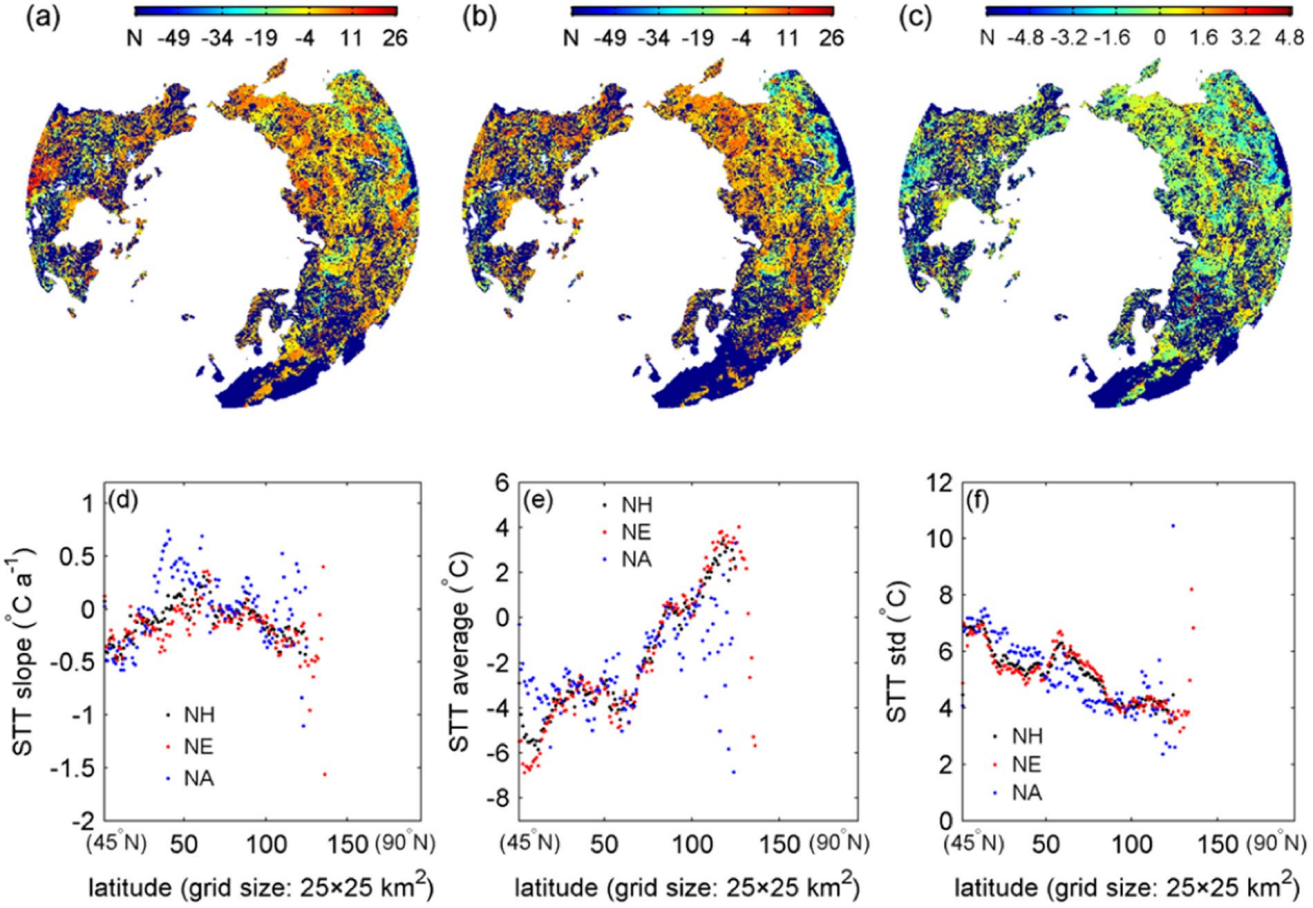
Illustration of the STT over the North Hemisphere between 2000 and 2001 (**a**) and between 2007 and 2008 (**b**) and STT slope (**c**) from 2000 to 2008 derived from the used dataset. Change of STT slope (**d**), STT average (**e**) and STT standard deviation (std) (**f**) in latitude over the North Hemisphere (NH), North America (NA), and North Eurasia (NE) (>45°N) from 2000 to 2008 derived from the used dataset, respectively. (**a**–**c**) are mapped at a 25 × 25 km^2^ resolution. The figure was created using *Matlab* ^38^.

The values of the grid-level STT, in terms of the prescribed temperature feature parameter of the maximum temperature relating to the highest “driving” percentage, are annually illustrated in Fig. 6a and b, which display the spatial characteristics of the temperature feature able of driving snow cover expansion across the NH. From the circumpolar Arctic to the temperate zone, the STT is decreasing in a whole sense. The slopes of this critic temperature feature in different grids from 2000 to 2008 are shown in Fig. 6c. Although there were some areas with extremely varying STTs, the slopes over the major parts of the NH proved to range from −1.6 °C/year to 1.6 °C/year (within 90% confidence level).

The latitudinal changes of the slope, average and std of the same STT are shown in Fig. 6d,e, and f, respectively. The three features varied in latitude in an approximate way for the NH, NA, and NE. The slope varied in latitude with an obvious turning, from increasing to decreasing. The average in latitude fluctuated but displayed an increasing trend toward the Arctic. The std in latitude also fluctuated but exhibited a decreasing trend when approaching the circumpolar, and this fits the snow-related phenomenon that high latitudes relate to fewer unstable scenarios than temperate zone, as shown in Fig. 1.

### Driving rule analysis

Apart from the layout of the maximum temperature-categorized SST as exposed above, many other details of the snow-driving rules can also be analyzed. For example, the OP days in latitude primarily exhibited the decreasing-to-increasing turnings, only with four different cases in the 18 curves (Fig. 5g–i). In comparison with Fig. 6d, it seems that its mainstream turning pattern shows an opposite interlink with the turning mode presented by the STT slope in latitude, and it, further, can be inferred that the product between the OP slope and STT slope in latitude can keep constant to some extent. In other words, the OP days in latitude might be in turn adapted by the temporal variations of the STT, which though needs to be tested with more reference data. It is expected that this full-of-uncertainty inference can elicit more studies regarding the more specific rules of temperature driving snow cover expansion from the Arctic.

As regards to the snow Do average in latitude (Fig. 2b, NH: r^2^ = 0.98, p < 0.001; NE: r^2^ = 0.97, p < 0.001; NA: r^2^ = 0.97, p < 0.001, respectively), it displayed a relatively smooth and linear change mode. The similar modes in latitude occurred to the snow Do std (Fig. 2c, NH: r^2^ = 0.78, p < 0.001; NE: r^2^ = 0.73, p < 0.001; NA: r^2^ = 0.76, p < 0.001), STT average (see Fig. 6e, NH: r^2^ = 0.70, p < 0.001; NE: r^2^ = 0.73, p < 0.001; NA: r^2^ = 0.31, p < 0.001), and STT std (Fig. 6f, NH: r^2^ = 0.59, p < 0.001; NE: r^2^ = 0.50, p < 0.001; NA: r^2^ = 0.57, p < 0.001). However, this does not mean that all of the local areas in the NH abide by this rule. This inference is evidenced by the Supplementary Fig. S4, in which east Europe proved to be the region with the most serious changes for all of the six temperature parameters. In other words, the snow cover expansions in east Europe may be driven by different temperature features. This inference is somehow consistent with the finding that the most drastic changes of snow cover phenology lie in east Europe^3^. The explanations are similar to those in the previous study^3^. That is, the snow phenology trend of this particular region is basically consistent with the observed long-term tendency of large-scale cooling phenomena of LST in winter across the mid-latitudes^32,33^.

The second unstable region in snow cover expansion is the NA. This inference is evidenced by the Supplementary Fig. S4, in which the NA also presented serious changes for most of the six temperature features. Different from east Europe that cannot compare to the NE in terms of area and, hence, showed no obvious disturbance to the whole pattern of the NE, the NA displayed this instability, as illustrated by the latitudinal section with fluctuated snow Do slopes in Fig. 2a. For such regional inconsistency in temperature driving snow cover expansion, more other kinds of environmental factors^6^ need to be introduced and examined to explain it. Moreover, the climate simulation models^30^ need also to put more attention to such special regions, e.g., central NH and east Europe, and more powerful modules capable of simulating the contrasting features of snow cover phenology in those special regions shall be developed to reduce the uncertainties in future climate projections.

The previous study indicated that contrasting snow cover phenology characteristics existed between the NH middle and high latitudes^3^, but the derived results concerning the statistical rules of temperature driving snow cover expansion in this study showed no such spatial modes. Does this mean that the mode of temperature driving snow cover expansion was less disturbed by the effect of Arctic warming^34^? This question needs further studies. After all, apart from temperature, the effect of precipitation, radiative energy and albedo on snow cover expansion must also exist, and future studies need to investigate how these energy budget anomalies and extreme events^35^ lead to the interannual variations of snow cover expansion and climate change. The increasing availability of long, high-resolution time-series of snow cover observations from satellites may help.

Last, but not least, this study briefly answered the question on “how” temperature driving snow cover expansion in the NH, not yet involving “why”. That is, the used statistical methods can help to achieve the new findings such as maximum temperature playing a leading role, but cannot help to reveal the driving mechanism. After all, snowfall is physically caused in specific atmospheric conditions involving radiative forcing^2^, air humidity, wind speed, air temperature^15^, terrain surface^36^, and other factors^1^. A more reliable way to expose this kind of mechanism is to use land surface process models^12,30^, which mean a lot of heavy researches in the future. Note that this does not weaken the significance of this study, the contributions of which can help to control and validate the snow-related functional modules of the assumed land surface models.

## Methods

### Data

This study required the data of two key parameters, i.e., snow Do and LST, to implement the induction of the statistical rules of temperature driving snow cover expansion. Given that accurate and continuous parameters facilitate revealing the statistical rules in a more reliable way, we used the SSM/I-SSMIS data (2000–2008)^24^ that can simultaneously provide the synchronous snow Do and LST parameters at the daily scale. The values of these two key features were derived based on the same set of remote sensing data that was acquired by the same passive microwave radiometric sensors onboard the Defense Meteorological Satellite Program (DMSP) polar-orbiting satellite^37^. This dataset, theoretically, can perform better than the other often-used snow datasets such as the reanalyzed dataset of daily snow depth published by the Canada Meteorological Center (CMC)^25^, the binary daily snow cover mask data generated from the Interactive Multi-sensor Snow and ice mapping system (IMS)^26^, the data of Northern Hemisphere weekly Snow Cover & sea ice Extent (NHSCE)^27^, the eight-day Level-3 snow cover fraction product (MOD10C2) output based on the MODerate resolution Imaging Spectroradiometer Satellite (MODIS)^28^ data, and the Snow Water Equivalent (SWE) data generated from the Near-real-time Ice and Snow Extent (NISE) dataset^29^. These datasets can output the parameter of snow Do only or the synchronous snow Do and LST parameters but at coarser temporal scales. That is, it is inappropriate to choose these datasets to implement this study.

In fact, the SSM/I-SSMIS data^24^ stretched over the period of 2000–2011, the monitoring of which came true based on the onboard F13 (1995–2009) and F17 (2006–now) instruments in succession. Because there were biases in the LST retrievals from the F13 and F17 brightness temperature^24^, particularly for some local regions, this study selected only the F13-derived data (2000–2008) in order to ensure the consistency of data for driving rule exploitation. The chosen dataset restricts the snow cover regions (grids) to the terrestrial area ranging between 45°N and 75°N, excluding the regions with permanent snow cover and ice like the Greenland. In addition, analysis of the driving rules requires inter-annual comparisons of snow cover phenology, whereas some regions might show snow covers in just one or two years. To avoid deriving the erroneous trends of snow development, this study was restricted to the stable snow cover regions where the snow covers must occur at least in five years during 2000–2008.

### Snow onset date identification and characteristics analysis

For the task of investigating how temperature drives snow cover expansion, Do is the most appropriate choice to reflect the snow cover expansion phenology. The detailed identification of the snow cover status was to seek the first five consecutive days on which snow was observed to cover the ground surface in the snow cover accumulation season^3,31^, and the first day within the sought five consecutive days was defined as the Do. Besides, note that for some areas in the NH, snow cover might occur in autumn or spring, unnecessarily in winter. Correspondingly, the snow onset dates in this study were determined by pursuing the snow cover status throughout the snow cover accumulation season^3,30^ – from the previous year (*t*-1) August to the current year (*t*) July, different from the traditional practice of seeking Do in winter (December–January–February) in the previous studies^31^.

The spatial and temporal characteristics of the snow cover expansion phenology (2000–2008) were quantified by calculating the slope of the Do series for each 25 × 25km^2^ grid^24^. The slope was calculated by using the linear regression analysis^32^. Then, the latitudinal variations of the snow Do slope, Do average and Do std were analyzed, and the results were expected to reflect the different performance of snow cover expansion for the different distances from the Arctic. The latitudinal changes of the three parameters were individually calculated by statistics of the related values for all of the grids at the same latitudes. Further, in order to reveal the distinctive driving rules possibly existing at different continents, the calculation was also deployed on the NA and NE separately.

### Derivation of the rules of temperature driving snow cover expansion

The specific rules of temperature driving snow cover expansion principally concerned what kinds of the LST feature parameters and how many days in advance readily leading to the snow cover status, namely, controlling the Do, for each grid in the NH. In this study, six statistics-sense LST feature parameters were derived, including average (T1), the largest difference (T2), std (T3), slope (T4), the minimum (T5) and the maximum (T6), from the serial LST data (2000–2008) for each NH grid. Since drive rather than link was highlighted in this study, the temporally-closest 30 days were prescribed as the temporal range in determining the OP^22^, which determines how many days in advance. The solution was to pursue the optimal correlation based on regression analysis^32^ between the LST feature parameter series and the Do values during 2000–2008 for each grid. The identified optimal case can reveal the secret about which of the prescribed LST feature parameters at how many days in advance drives the snow expansion in each NH grid. The value of that LST feature parameter relating to the optimal case is assigned to the STT variable.

The spatial and temporal characteristics of the different driving rules individually presented by the 25 × 25 km^2^ grids^24^ over the NH were investigated. Specifically, the percentage, correlation coefficient (r^2^) and OP days for each of the prescribed LST feature parameters were mapped and compared in statistics. Then, the latitudinal variations of the percentage, r^2^ and OP days for each of the LST feature parameters were analyzed by fitting them into quadratic polynomial functions, with each of these parameters assumed as the dependent variable and latitude as the independent variable. The results were expected to reflect the latitudinal variation modes of the driving rules. The latitudinal variations of the above-concerned three feature parameters were individually derived by statistics of the related parameter values for all of the grids at the same latitudes. Further, in order to expose the distinct driving rules possibly existing at different continents, the same operations were separately deployed onto the NA and NE.

To facilitate better understanding of the derived rules about how temperature driving snow cover expansion, the spatial and temporal characteristics of the STT parameter, illustratively in terms of the maximum temperature, were examined by calculating its slope (2000–2008) for each 25 × 25km^2^ grid^24^ in the NH. The slope was calculated using the linear regression analysis^32^. The latitudinal variation curves of the STT slope, STT average and STT std were generated, and the results were analyzed to explain the different modes about the driver of snow cover expansion from the Arctic. The latitudinal changes of the STT variable were calculated in the same way as handling the parameters of percentage, r^2^ and OP days in the above process of identifying the driving rules. These operations were deployed onto the NA and NE separately, and this may help to better explain the different driving rules shown by the two continents.

### Data Statement

Readers can access the data via contact to the authors.

## Electronic supplementary material

Supplementary information
