# Supplementary material for: Maximum temperature drove snow cover expansion from the Arctic, 2000–2008
Source: Sci Rep. 2017 Nov 8;7:15090. doi: 10.1038/s41598-017-15397-3 (PMC5678124; doi:10.1038/s41598-017-15397-3)
Supplement: Supplementary file 1 — Supplementary information [file 41598_2017_15397_MOESM1_ESM.doc]

**Maximum temperature drove snow cover expansion from the Arctic, 2000-2008**

Yi Lin ^1, *^, Miao Jiang ^2^

^1^ School of Earth and Space Sciences, Peking University, Beijing 100871, China

^2^ Institute of Mineral Resources Research, China Metallurgical Geology Bureau, Beijing 100025, China

^*^ Correspondence, Email: yi.lin@pku.edu.cn

**Supplementary results:**

The resulting Do dates over the NH during 2001–2007 are annually shown in Supplementary Fig S1. Combined with Fig. 1a and 1b, the serial Do maps can intuitively demonstrate the spatial characteristics of snow cover expansion from the Arctic during 2000–2008. That is, although there were some regional fluctuations, the integral pattern kept being briefly consistent. The Alaska and Russian Far East exhibited the earliest snow onsets, while the West and Middle Europe were just on the opposite. From the circumpolar to the temperate zone, the snow onset dates were gradually delayed in a whole sense.


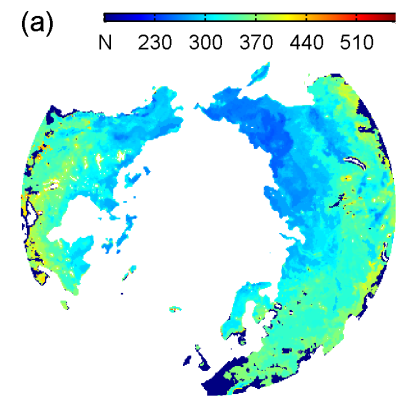

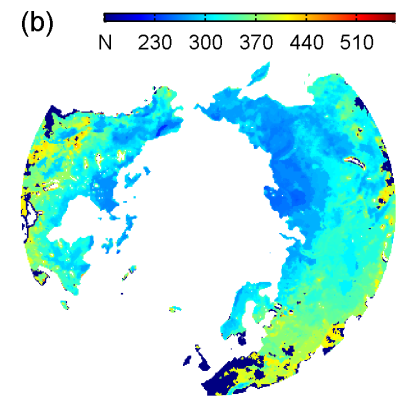

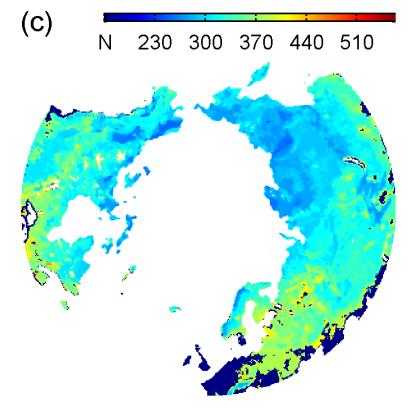


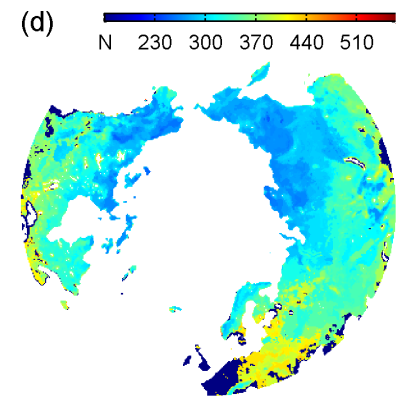

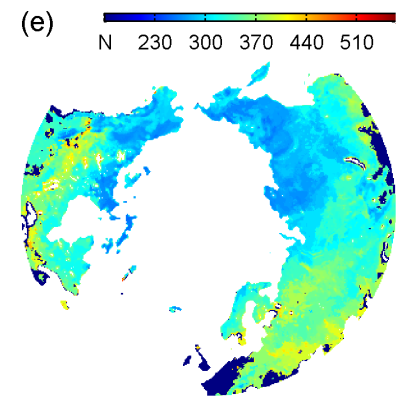

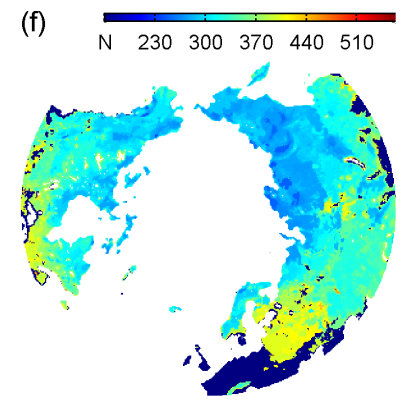


**Supplementary Figure S1. Snow onset date (Do) over the North Hemisphere between 2001 and 2002 (a), between 2002 and 2003 (b), between 2003 and 2004 (c), between 2004 and 2005 (d), between 2005 and 2006 (e), and between 2006 and 2007 (f) derived from the used data set.** (**a–f**) are mapped at a 25×25 km^2^ resolution. The figure was created using *Matlab*^37^.

The fittings of the dependent variables (percentage, r^2^ and OP days) and the independent variables (the six LST features) into quadratic polynomial functions are shown in Supplementary Fig. S2. The resulting feature parameters (min, max and median), polynomial coefficients (a, b and c) and mode of the quadratic polynomial fitting are listed in Supplementary Table T1. Based on these parameters, the turning points and variation mode in terms of latitude can be determined. Along with latitude increasing, \/ denotes first decreasing and then increasing, /\ is on the opposite, / indicates monotonously increasing, and \ is just on the opposite.


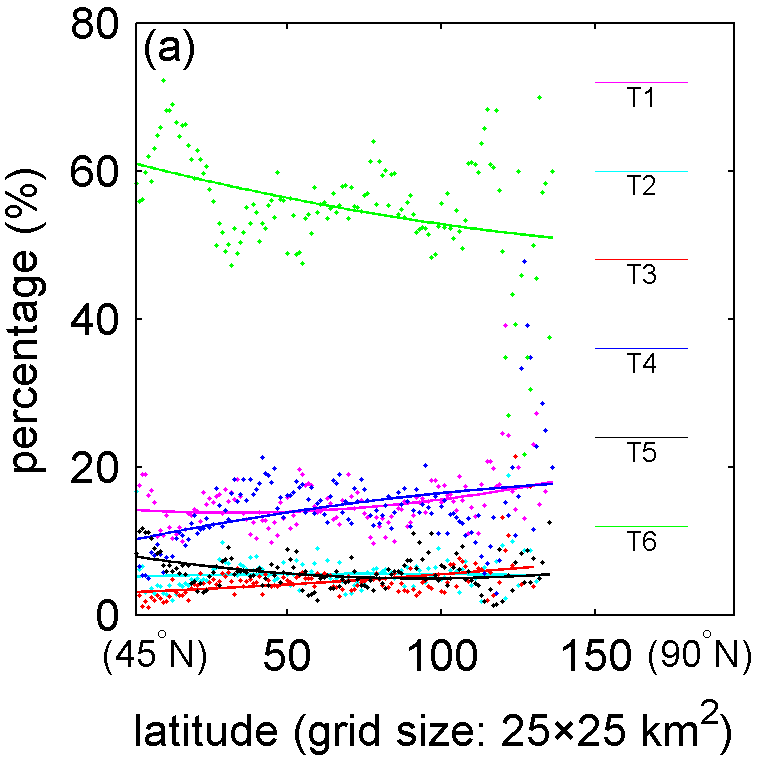

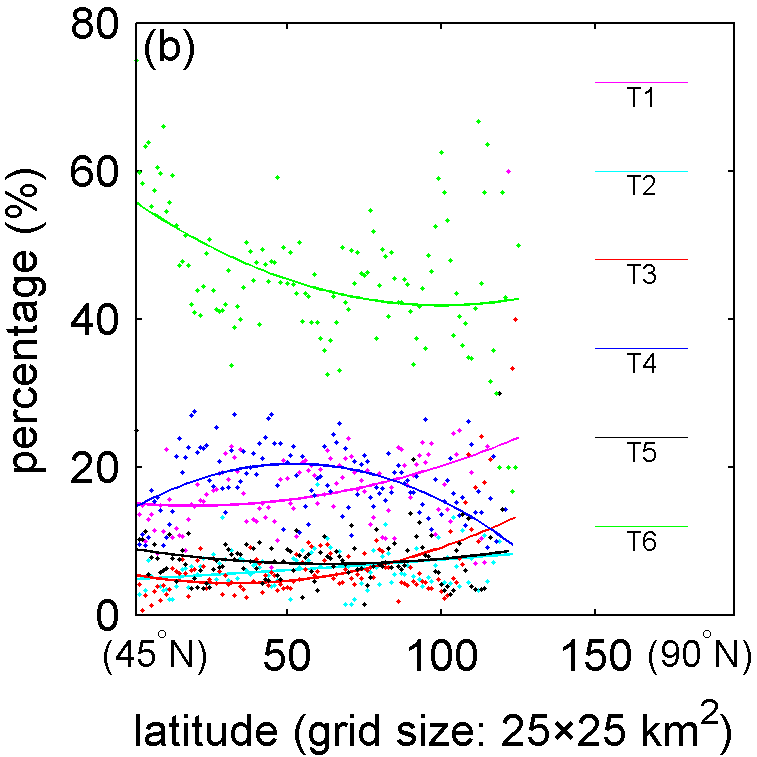

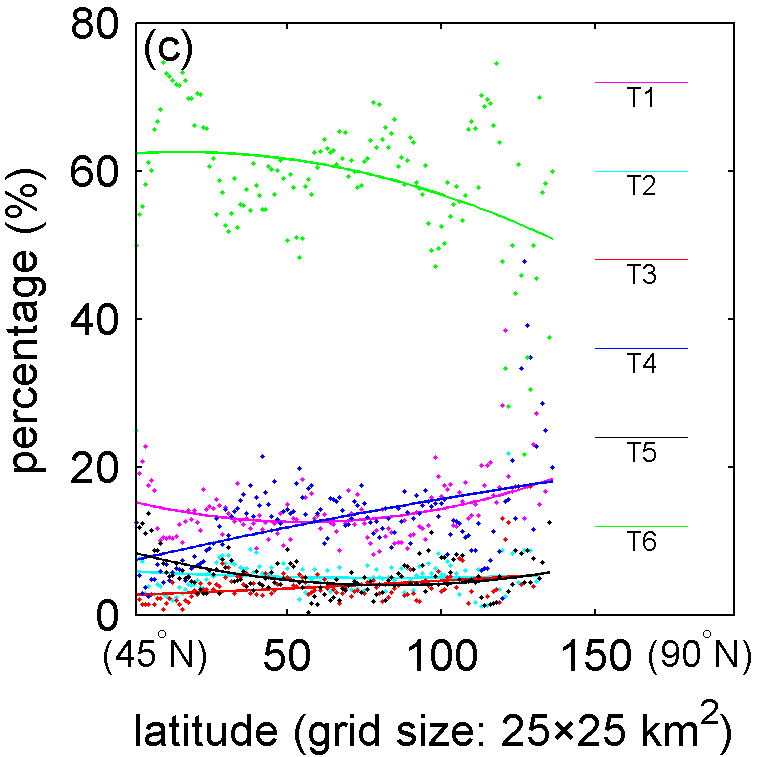


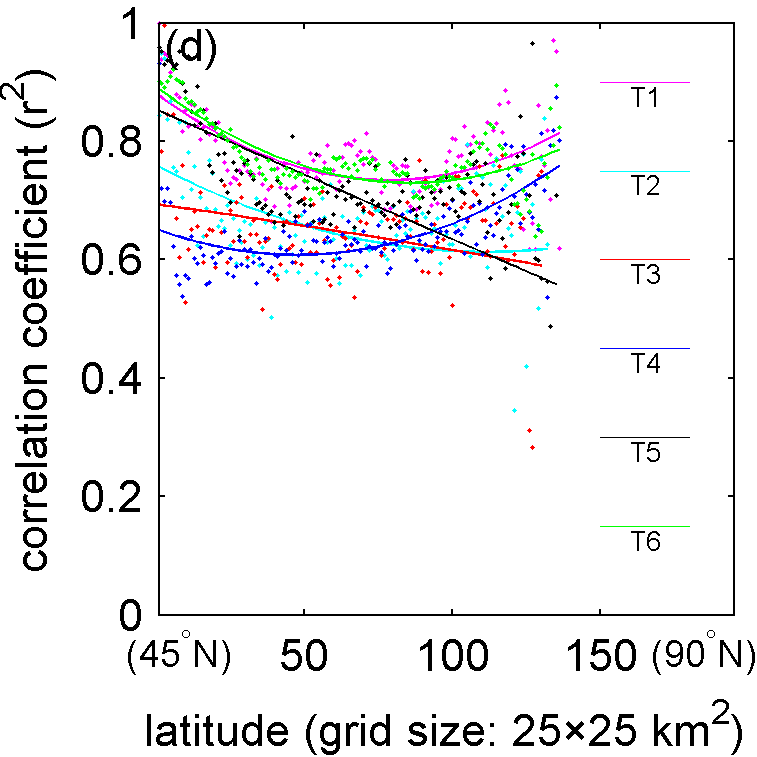

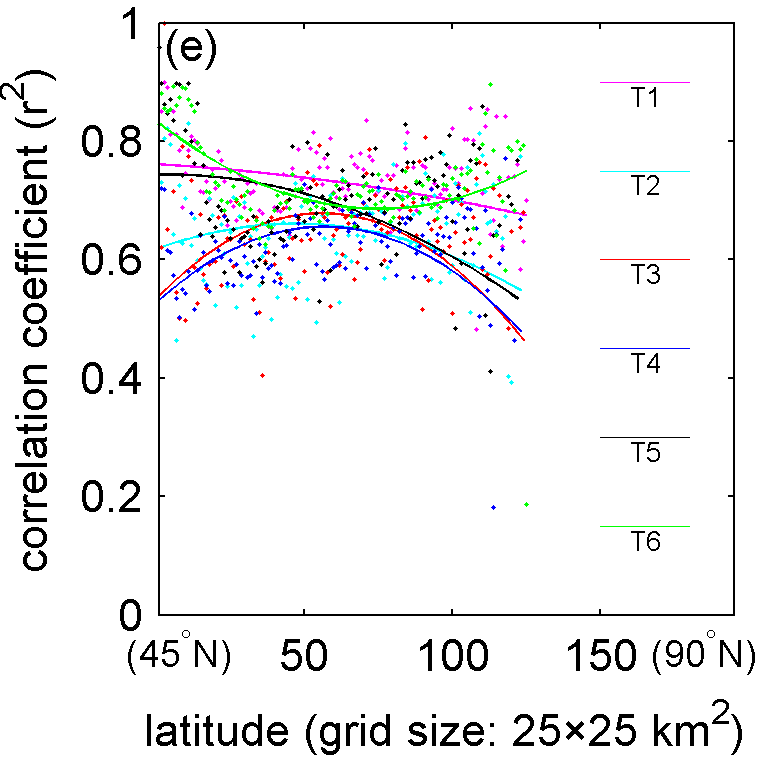

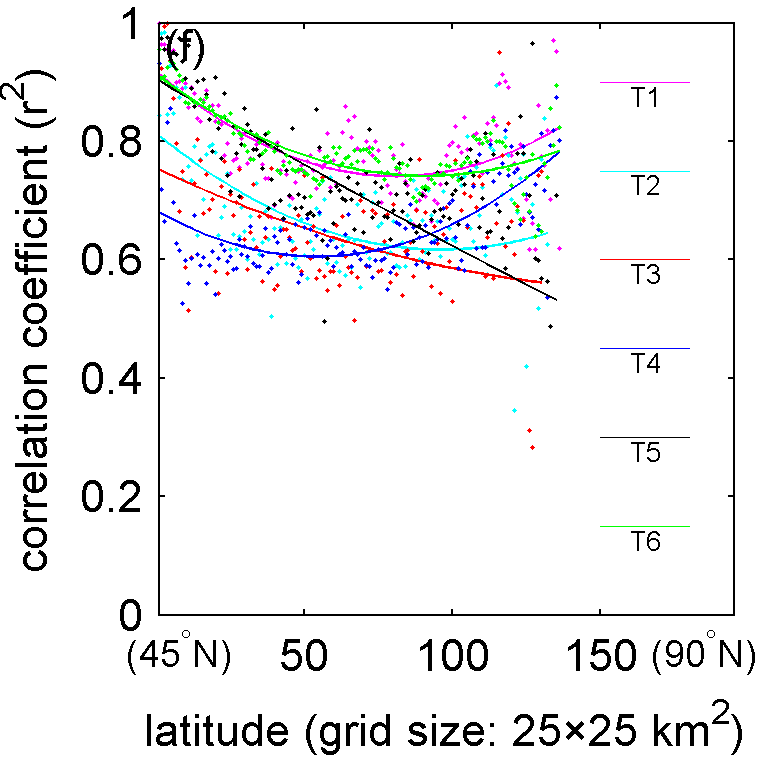


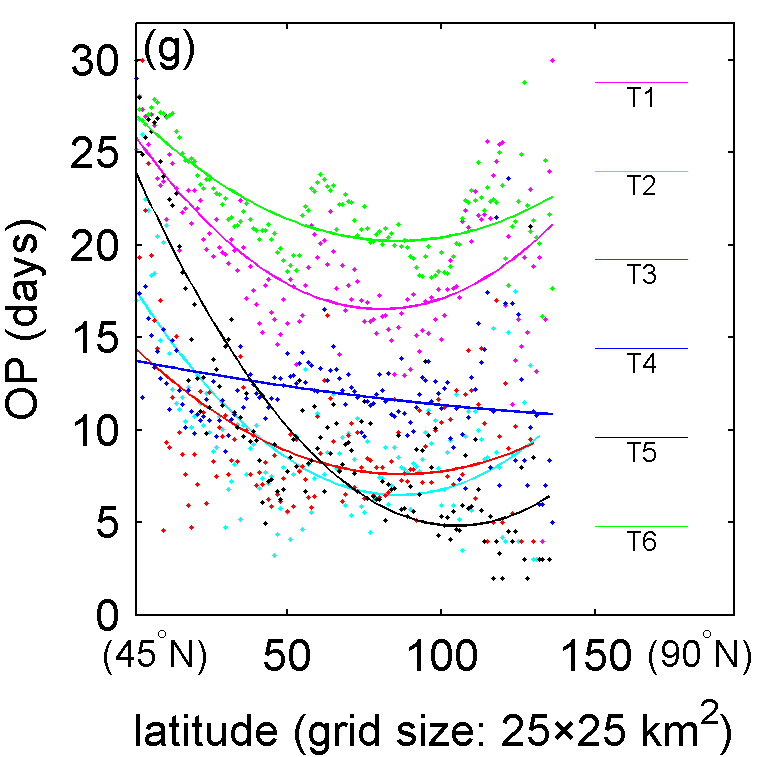

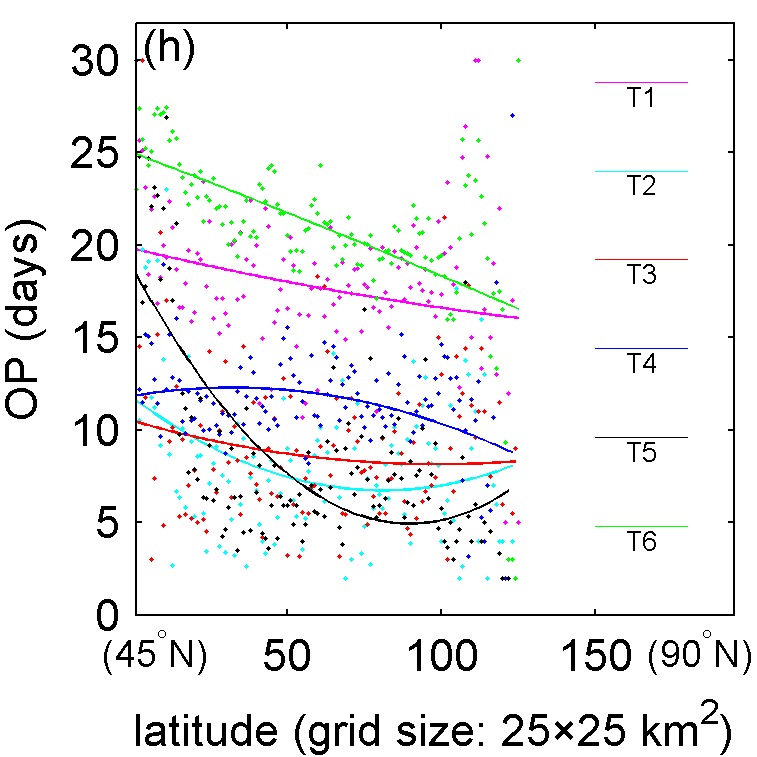

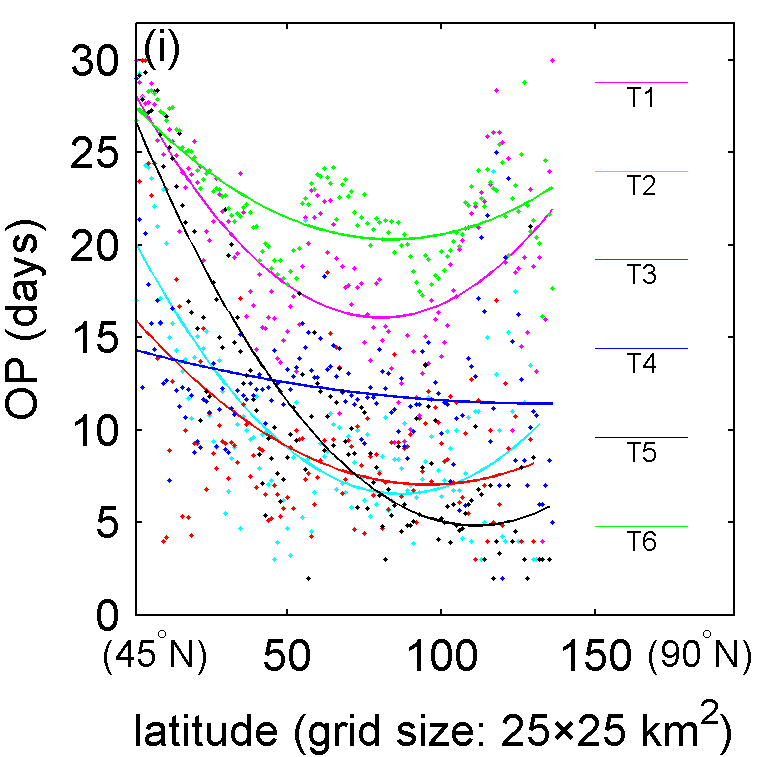


**Supplementary Figure S2. Quadratic polynomial fitting of percentage (a), correlation coefficient (R^2^) (b) and OP days (c) of the STTimal correlation cases in latitude for the six types of temperature parameters.** (**a, d and g**) North Hemisphere (NH), (**b, e and h**) North America (NA), and (**c, f and i**) North Eurasia (NE). The figure was created using *Matlab*^37^.

**Supplementary Table T1. The feature parameters (min, max and median), polynomial coefficients (a, b and c) and mode of the quadratic polynomial fitting corresponding to Supplementary Figure S2, individually in terms of different temperature feature types (T1, T2, T3, T4, T5 and T6) and different statistical features (percentage, R^2^ and OP days).**

| feature | statistics | min | max | median | a | b | c | mode |
| --- | --- | --- | --- | --- | --- | --- | --- | --- |
|  | percentage | 1 | 136 | 32 | 3.80E-04 | -0.0243 | 14.1761 | \/ |
| T1 | R^2^ | 1 | 136 | 78 | 2.39E-05 | -0.0037 | 0.8811 | \/ |
|  | OP days | 1 | 136 | 80 | 1.50E-03 | -0.2369 | 26.0588 | \/ |
|  | percentage | 1 | 132 | 75 | -6.43E-05 | 0.0097 | 5.1793 | /\ |
| T2 | R^2^ | 1 | 132 | 111 | 1.19E-05 | -0.0026 | 0.7601 | \/ |
|  | OP days | 1 | 132 | 86 | 1.50E-03 | -0.2625 | 17.7832 | \/ |
|  | percentage | 2 | 130 | -117 | 7.25E-05 | 0.0169 | 3.058 | / |
| T3 | R^2^ | 2 | 130 | -445 | -7.80E-07 | -0.0007 | 0.6936 | \ |
|  | OP days | 2 | 130 | 87 | 9.00E-04 | -0.159 | 14.5222 | /\ |
|  | percentage | 1 | 136 | 196 | -2.16E-04 | 0.0849 | 10.1202 | / |
| T4 | R^2^ | 1 | 136 | 48 | 1.93E-05 | -0.0018 | 0.6521 | \/ |
|  | OP days | 1 | 136 | 209 | 1.00E-04 | -0.0317 | 13.7523 | \ |
|  | percentage | 1 | 135 | 94 | 3.38E-04 | -0.0638 | 7.9257 | \/ |
| T5 | R^2^ | 1 | 135 | 6140 | 1.80E-07 | -0.0022 | 0.8538 | \ |
|  | OP days | 1 | 135 | 105 | 1.80E-03 | -0.3706 | 24.2935 | \/ |
|  | percentage | 1 | 136 | 228 | 2.31E-04 | -0.1056 | 61.0427 | \ |
| T6 | R^2^ | 1 | 136 | 86 | 2.22E-05 | -0.0038 | 0.8923 | \/ |
|  | OP days | 1 | 136 | 86 | 9.00E-04 | -0.1619 | 27.1465 | \/ |
|  | percentage | 2 | 125 | 20 | 0.0008 | -0.0324 | 15.0644 | \/ |
| T1 | R^2^ | 2 | 125 | -49 | -3.10E-06 | -0.0003 | 0.7614 | \ |
|  | OP days | 2 | 125 | 254 | 1.00E-04 | -0.0399 | 19.7988 | \ |
|  | percentage | 2 | 123 | -237 | 0 | 0.0218 | 4.8197 | / |
| T2 | R^2^ | 2 | 123 | 47 | 1.95E-05 | 0.0018 | 0.6173 | \/ |
|  | OP days | 2 | 123 | 81 | 8.00E-04 | -0.1228 | 11.7062 | \/ |
|  | percentage | 2 | 124 | 33 | 0.0011 | -0.0699 | 5.4095 | \/ |
| T3 | R^2^ | 2 | 124 | 56 | -4.65E-05 | 0.0052 | 0.5326 | /\ |
|  | OP days | 2 | 124 | 99 | 2.00E-04 | -0.0473 | 10.4772 | \/ |
|  | percentage | 2 | 123 | 52 | -0.0022 | 0.2292 | 14.392 | /\ |
| T4 | R^2^ | 2 | 123 | 57 | -4.02E-05 | 0.0046 | 0.5261 | /\ |
|  | OP days | 2 | 123 | 32 | -4.00E-04 | 0.0276 | 11.8337 | /\ |
|  | percentage | 1 | 122 | 63 | 0.0005 | -0.0639 | 8.9037 | \/ |
| T5 | R^2^ | 1 | 122 | 4 | -1.50E-05 | 0.0001 | 0.7436 | /\ |
|  | OP days | 1 | 122 | 90 | 1.70E-03 | -0.3078 | 18.7457 | \/ |
|  | percentage | 1 | 125 | 100 | 0.0014 | -0.2835 | 55.9914 | \/ |
| T6 | R^2^ | 1 | 125 | 75 | 2.62E-05 | -0.0039 | 0.8345 | \/ |
|  | OP days | 1 | 125 | -841 | 0.00E+00 | -0.0627 | 24.9729 | / |
|  | percentage | 1 | 136 | 55 | 8.94E-04 | -0.0992 | 15.2875 | \/ |
| T1 | R^2^ | 1 | 136 | 81 | 2.71E-05 | -0.0044 | 0.9179 | \/ |
|  | OP days | 1 | 136 | 81 | 1.90E-03 | -0.306 | 28.3857 | \/ |
|  | percentage | 1 | 132 | 81 | 1.32E-04 | -0.0214 | 5.8565 | \/ |
| T2 | R^2^ | 1 | 132 | 96 | 2.13E-05 | -0.0041 | 0.8134 | \/ |
|  | OP days | 1 | 132 | 87 | 1.80E-03 | -0.3202 | 20.4268 | \/ |
|  | percentage | 2 | 130 | -165 | 4.43E-05 | 0.0146 | 2.7004 | / |
| T3 | R^2^ | 2 | 130 | 174 | 6.83E-06 | -0.0024 | 0.755 | \ |
|  | OP days | 2 | 130 | 96 | 1.00E-03 | -0.1895 | 16.1097 | \/ |
|  | percentage | 1 | 136 | 374 | -1.29E-04 | 0.0963 | 7.312 | / |
| T4 | R^2^ | 1 | 136 | 54 | 2.65E-05 | -0.0029 | 0.6837 | \/ |
|  | OP days | 1 | 136 | 133 | 2.00E-04 | -0.044 | 14.3419 | \/ |
|  | percentage | 2 | 135 | 83 | 6.41E-04 | -0.1066 | 8.4168 | \/ |
| T5 | R^2^ | 2 | 135 | 936 | 1.59E-06 | -0.003 | 0.9054 | \ |
|  | OP days | 2 | 135 | 111 | 1.80E-03 | -0.4002 | 27.0798 | \/ |
|  | percentage | 1 | 136 | 16 | -8.13E-04 | 0.0261 | 62.3316 | /\ |
| T6 | R^2^ | 1 | 136 | 91 | 2.08E-05 | -0.0038 | 0.9138 | \/ |
|  | OP days | 1 | 136 | 84 | 1.00E-03 | -0.1736 | 27.575 | \/ |

The resulting STTs over the NH during 2001–2007 are annually displayed in Supplementary Fig S3. Combined with Fig. 6a and 6b, the serial STT maps can intuitively demonstrate the spatial characteristics of the temperature capable of driving snow cover expansion from the Arctic during 2000–2008. Specifically, although there were some regional fluctuations, the integral mode of the STTs in Asia was increasing along with the latitude increasing; instead, the mode in Europe was different in terms of longitude, i.e., the central Europe related to high STT while the east Europe to low STT; the STT mode in north America was spatially inconsistent.


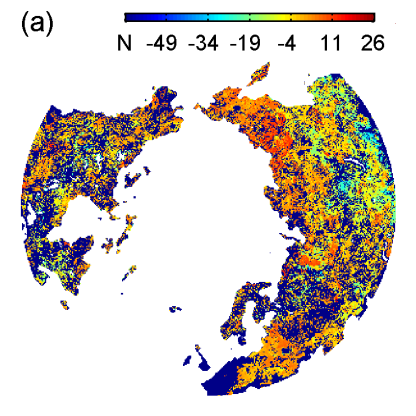

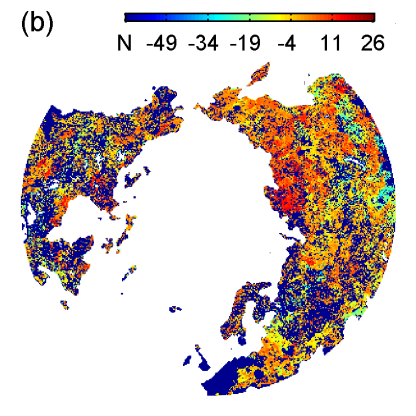

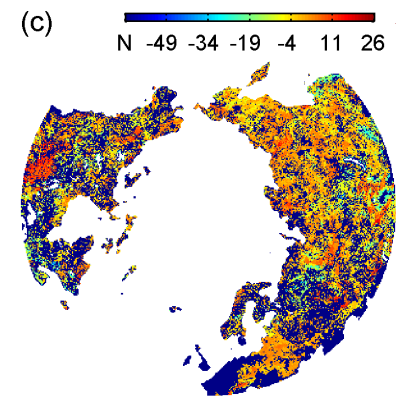


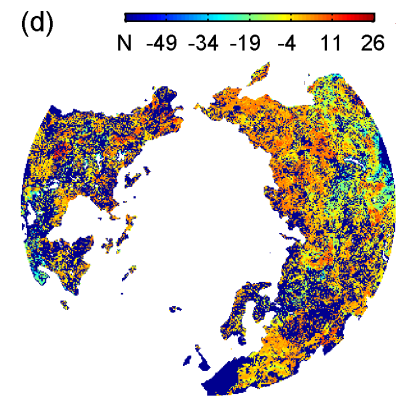

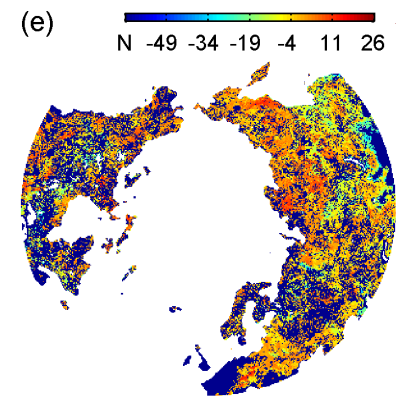

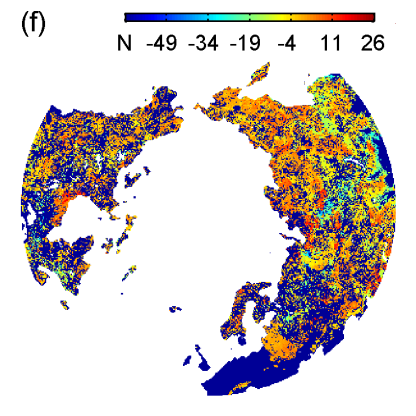


**Supplementary Figure S3. STT over the North Hemisphere between 2001 and 2002 (a), between 2002 and 2003 (b), between 2003 and 2004 (c), between 2004 and 2005 (d), between 2005 and 2006 (e), and between 2006 and 2007 (f) derived from the used data set.** (**a–f**) are mapped at a 25×25 km^2^ resolution. The figure was created using *Matlab*^37^.

The results of the grid-level correlations between the Do series and the related values of the six temperature parameters over the NH during 2000–2008 are shown in Supplementary Fig S4. It is obvious that the sixth temperature parameter (the maximum) took a leading role, as evidenced in Fig. 3 and 4, while the fifth temperature parameter (the minimum) worked the least. The second and third parameters (the largest difference and std) helped quite less either, mainly working in the east Europe. The first and fourth parameters (mean and slope) functioned in higher ratios, but they showed different spatial mode in the NE, i.e., the effects of the first parameter are almost evenly distributed over the NE while the effects of the fifth parameter concentrate into the east Europe. In the NA, their spatial distributions were both scattered.


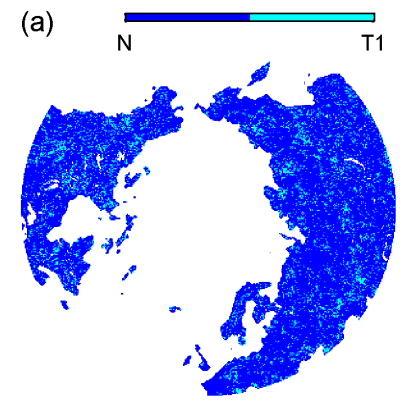

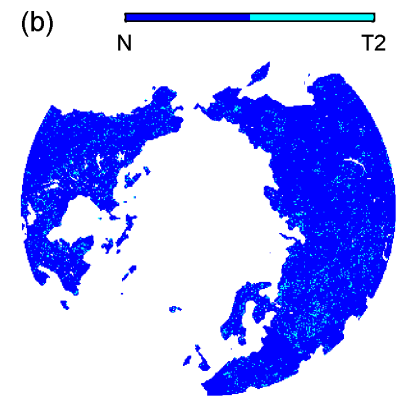

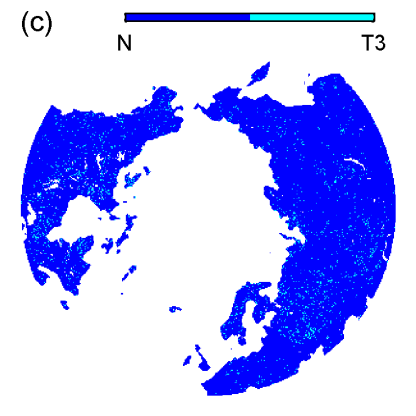


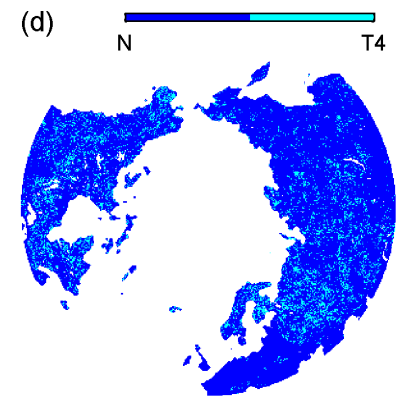

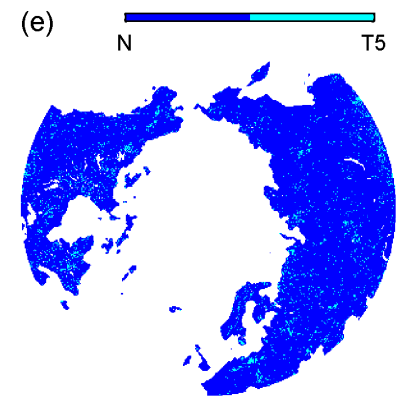

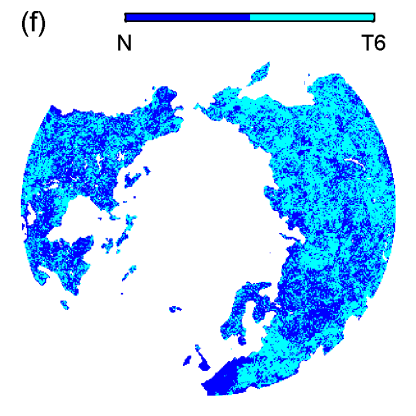


**Supplementary Figure S4. Spatial distributions of the best correlations at the grid level with different temperature parameters: mean (a), the largest difference (b), std (c), slope (d), the minimum (e), and the maximum (f) derived from the used data set.** (**a–f**) are mapped at a 25×25 km^2^ resolution. The figure was created using *Matlab*^37^.
